# Assessment of Metformin induced changes in cardiac redox state using hyperpolarized[1-^13^C]pyruvate

**DOI:** 10.1186/1532-429X-18-S1-O24

**Published:** 2016-01-27

**Authors:** Andrew Lewis, Chloe McCallum, Jack J Miller, Oliver Rider, Stefan Neubauer, Lisa Heather, Damian J Tyler

**Affiliations:** University of Oxford, Oxford, UK

## Background

Metformin improves cardiovascular outcomes in diabetes, but its mechanism of action is controversial. Recent evidence suggests that Metformin reduces gluconeogenesis by altering hepatic redox state. Whether Metformin also alters cardiac redox state and metabolism is unknown, in part because of the difficulty in measuring cardiac metabolism *in vivo*. Hyperpolarized [1-^13^C]pyruvate magnetic resonance spectroscopy can assess the redox coupled interconversion of pyruvate and lactate with spatial localization to the heart. We therefore used hyperpolarized [1-^13^C]pyruvate magnetic resonance spectroscopy to investigate the effects of acute and chronic metformin treatment upon cardiac and hepatic metabolism.

## Methods

In the first study, male Wistar rats (n = 10 per group) were given an intravenous infusion of either 50 mg Metformin or saline. Hyperpolarized [1-^13^C]pyruvate spectroscopy was performed 45 minutes later, with slice selective spectra acquired from two axial slabs covering the heart and liver using a 7T preclinical MR system and a volume ^13^C transmit / two-channel surface receive RF coil. We next tested the effects of longer term Metformin treatment (4 weeks) in both control rats and rats with a high-fat feeding and streptozotocin induced model of type II diabetes (n = 6-8 per group). We again used hyperpolarized [1-^13^C]pyruvate spectroscopy to assess cardiac and hepatic metabolism, and also performed echocardiography to assess cardiac diastolic function.

## Results

**Figure 1:**
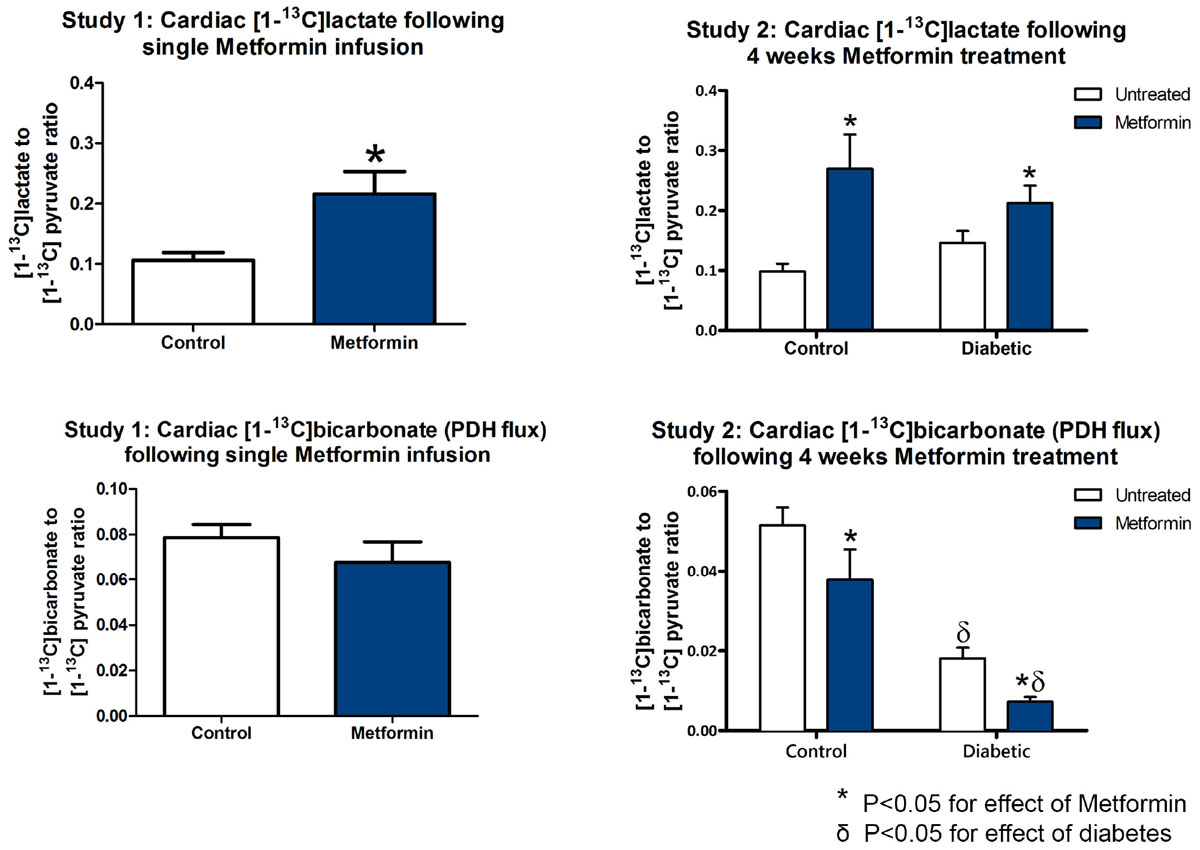
Figure 1

A single Metformin infusion significantly increased the cardiac [1-^13^C]lactate:[1-^13^C]pyruvate ratio (0.22 ± 0.04 versus 0.11 ± 0.01, P < 0.05, Figure 1) without changing pyruvate dehydrogenase flux ([1-^13^C]bicarbonate:[1-^13^C]pyruvate ratio 0.07 ± 0.009 versus 0.08 ± 0.006, P = ns). Longer term Metformin treatment for 4 weeks also increased the cardiac [1-^13^C]lactate:[1-^13^C]pyruvate ratio in both control rats (0.26 ± 0.01 versus 0.10 ± 0.01, P < 0.05) and in diabetic rats (0.21 ± 0.03 versus 0.14 ± 0.02, P < 0.05). Surprisingly, Metformin also reduced cardiac pyruvate dehydrogenase flux in both control (0.04 ± 0.08 versus 0.05 ± 0.004, P < 0.05) and diabetic rats (0.007 ± 0.001 versus 0.02 ± 0.003, P < 0.05). Similar changes in both [1-^13^C]lactate and [1-^13^C]bicarbonate production were present in the liver. Diabetes was associated with diastolic but not systolic dysfunction (E/E' ratio 18 ± 1.4 versus 15 ± 0.8, P < 0.05), which was not changed by Metformin treatment (E/E' ratio 17 ± 1.2, versus 18 ± 1.4, P = ns).

## Conclusions

Both acute and chronic metformin treatment significantly increased the cardiac [1-^13^C]lactate:[1-^13^C]pyruvate ratio, likely reflecting a shift in cardiac redox state. These findings suggest that hyperpolarized 1-^13^C]pyruvate magnetic resonance spectroscopy is sensitive to Metformin induced changes in redox biology and can identify novel cardiac metabolic effects of this commonly prescribed drug.

